# The influence of patient characteristics on the alarm rate in intensive care units: a retrospective cohort study

**DOI:** 10.1038/s41598-022-26261-4

**Published:** 2022-12-16

**Authors:** Zeena-Carola Sinno, Denys Shay, Jochen Kruppa, Sophie A.I. Klopfenstein, Niklas Giesa, Anne Rike Flint, Patrick Herren, Franziska Scheibe, Claudia Spies, Carl Hinrichs, Axel Winter, Felix Balzer, Akira-Sebastian Poncette

**Affiliations:** 1 Charité – Universitätsmedizin Berlin, corporate member of Freie Universität Berlin and Humboldt-Universität zu Berlin, Institute of Medical Informatics, Charitéplatz 1, 10117 Berlin, Germany; 2 Harvard T.H. Chan School of Public Health, Department of Epidemiology, Boston, MA USA; 3 Hochschule Osnabrück, University of Applied Sciences, Osnabrück, Germany; 4 Charité – Universitätsmedizin Berlin, corporate member of Freie Universität Berlin and Humboldt-Universität zu Berlin, Department of Anesthesiology and Intensive Care Medicine, Charitéplatz 1, 10117 Berlin, Germany; 5 Berlin Institute of Health at Charité – Universitätsmedizin Berlin, Core Facility Digital Medicine and Interoperability, Charitéplatz 1, 10117 Berlin, Germany; 6 Charité – Universitätsmedizin Berlin, corporate member of Freie Universität Berlin and Humboldt-Universität zu Berlin, Department of Neurology, Charitéplatz 1, 10117 Berlin, Germany; 7 NeuroCure Clinical Research Center, Charitéplatz 1, 10117 Berlin, Germany; 8 Charité – Universitätsmedizin Berlin, corporate member of Freie Universität Berlin and Humboldt-Universität zu Berlin, Department of Nephrology and Medical Intensive Care, Charitéplatz 1, 10117 Berlin, Germany; 9 Charité – Universitätsmedizin Berlin, corporate member of Freie Universität Berlin and Humboldt-Universität zu Berlin, Department of Surgery, Charitéplatz 1, 10117 Berlin, Germany

**Keywords:** Outcomes research, Translational research, Health care

## Abstract

Intensive care units (ICU) are often overflooded with alarms from monitoring devices which constitutes a hazard to both staff and patients. To date, the suggested solutions to excessive monitoring alarms have remained on a research level. We aimed to identify patient characteristics that affect the ICU alarm rate with the goal of proposing a straightforward solution that can easily be implemented in ICUs. Alarm logs from eight adult ICUs of a tertiary care university-hospital in Berlin, Germany were retrospectively collected between September 2019 and March 2021. Adult patients admitted to the ICU with at least 24 h of continuous alarm logs were included in the study. The sum of alarms per patient per day was calculated. The median was 119. A total of 26,890 observations from 3205 patients were included. 23 variables were extracted from patients' electronic health records (EHR) and a multivariable logistic regression was performed to evaluate the association of patient characteristics and alarm rates. Invasive blood pressure monitoring (adjusted odds ratio (aOR) 4.68, 95%CI 4.15–5.29, p < 0.001), invasive mechanical ventilation (aOR 1.24, 95%CI 1.16–1.32, p < 0.001), heart failure (aOR 1.26, 95%CI 1.19–1.35, p < 0.001), chronic renal failure (aOR 1.18, 95%CI 1.10–1.27, p < 0.001), hypertension (aOR 1.19, 95%CI 1.13–1.26, p < 0.001), high RASS (aOR 1.22, 95%CI 1.18–1.25, p < 0.001) and scheduled surgical admission (aOR 1.22, 95%CI 1.13–1.32, p < 0.001) were significantly associated with a high alarm rate. Our study suggests that patient-specific alarm management should be integrated in the clinical routine of ICUs. To reduce the overall alarm load, particular attention regarding alarm management should be paid to patients with invasive blood pressure monitoring, invasive mechanical ventilation, heart failure, chronic renal failure, hypertension, high RASS or scheduled surgical admission since they are more likely to have a high contribution to noise pollution, alarm fatigue and hence compromised patient safety in ICUs.

## Introduction

Staff and patients in intensive care units (ICU) are confronted with an excessive amount of monitoring alarms on a daily basis^1,2^. Although alarming ICU staff of a deviation of a vital parameter is necessary in the context of delivering intensive care^3,4^, the majority of the alarms in the ICU are reported as false positive, caused by patient movement or poor skin contact of electrodes for example, and are often clinically non-actionable^5–10^. Most of these false alarms require no clinical intervention but cause disruption in the healthcare providers’ workflow^2,11^. This can lead to alarm fatigue, defined by Sendelbach and Funk^12^ as a sensory overload resulting from of the large number of particularly false alarms, and can have severe adverse effects on both the ICU staff and patients. For healthcare providers, these include decreased work satisfaction, reduced concentration, high stress levels and burnout^13^. For patients, it can lead to sleep disturbance^14–16^, higher rate of delirium^17,18^, and delayed recovery^13,14^. Alarm fatigue can even lead to life-threatening events and death. In fact, 98 alarm-related incidents between 2009 and 2012 were reported by the Joint Commission, an organization that evaluates and accredits healthcare institutions, 80 of which resulted in patient death^19^. Consequently, the ECRI Institute listed alarm-related hazards among their Top 10 Health Technology Hazards for the year 2020^20^.

In the literature, approaches to reduce the high rate of alarms in the ICU are being discussed. These include the implementation of nurse workshops^21^ to raise nurses’ awareness and offer solutions for better ‘alarm hygiene’, the development of algorithms^22^ and machine learning models, as well as the combination of different sensor waves^23^. However, to our knowledge, these approaches have not yet been integrated into the clinical routine, most likely to prevent suppression of crucial life-saving alarms, which might entail legal ramifications.

So far, research has not involved patient-specific features in the study of alarms and mostly relied on descriptive analysis or the suggestion of potential solutions to combat alarm fatigue. The aim of this study is to identify patient characteristics that influence the alarm rate in the ICU, particularly characteristics significantly associated with a high alarm rate.

## Methods

All collected data were stored on servers within the hospital’s network. Data extraction, data processing and statistical analysis were conducted using RStudio version 1.3.1093 and R version 4.0.3.

### Alarm logs

Every patient in the ICU is connected to a standard monitoring device that monitors their vital parameters; heart rate, blood pressure, SpO2 for instance. The alarms issued from these monitoring devices are mostly related to the patient's vital parameters (deviance from a predefined limit; for example, if a patient's SpO2 were below the predefined limit of 90%, an alarm would sound).

We extracted the alarm logs from monitoring devices of eight ICUs (on three different campuses) of a German tertiary care university hospital in Berlin (discontinuously) from September 2019 until March 2021. At the time of the study, the monitors in use in the included ICUs were part of the Philips IntelliVue patient monitoring system. Three of these ICUs are surgical, three are internal, and two are neurological/neurosurgical. The raw alarm logs consist of entries with information on the respective bed the alarm was issued from, the reason (value higher or lower than a threshold, pauses, technical alarms) and date/time of the alarm. Technical alarms are not related to the patient's clinical condition and were not included in this study. Similar data was published by Poncette et al.^24^.

Alarms issued from other devices such as infusion pumps, other medical devices, or refrigerators are mostly technical alarms, and concern battery level, obstruction of flow, device temperature, or drug levels (syringe empty etc.) for instance and were not part of this study.

### Patient inclusion criteria

Patients identified through the alarm logs were included if they were 18 years of age or older and if a minimum of 24 h of alarm data was available. During the study period, the hospital was gradually updating the electronic health records to a newer software and database, only patients from the new database (COPRA System GmbH) were included.

### Variable selection

The patient characteristics included in the analysis are demographic data (age, body mass index (BMI), sex), clinical scores to represent the clinical condition and severity of illness of the patients (Sequential Organ Failure Assessment (SOFA) score, Glasgow Coma Scale (GCS), Simplified Acute Physiology Score (SAPS) II, Confusion Assessment Method for the ICU (CAM-ICU), Richmond Agitation Sedation Scale (RASS), admission type and pre-existing conditions (Charlson Comorbidity Index (CCI), and presence of coronary heart disease, hypertension, asthma, chronic obstructive pulmonary disease, diabetes, heart failure, chronic kidney failure) as well as characteristics regarding ICU healthcare utilization (if mechanical ventilation, extracorporeal membrane oxygenation (ECMO), dialysis were received, if the patient was isolated, and if the patient had a central venous catheter in place).

### Extraction of patient characteristics

The above-mentioned patient characteristics were extracted from patients’ electronic health records. For data on ICU healthcare utilization, we extracted the associated timestamps (e.g., for the central line: the dates of placement and removal of the central line). If no data was available for a certain day, we considered that the patient did not receive the corresponding intervention. The BMI was calculated from the height and weight of a patient. The pre-existing conditions and each subscore of the CCI were extracted using ICD-10 GM codes. The CCI was then calculated for each patient. For mechanical ventilation, we extracted the intervals in which each patient received an airway device (Magill tube, face mask etc.). The airway devices were then categorized as invasive or non-invasive.

### Data engineering

The alarm logs were cleaned; only entries describing alarm start were selected, the alarms were categorized in groups depending on the origin of the alarm (electrocardiogram (ECG), ventilator etc.), as previously described^25^. Only alarms regarding ECG, invasive and non-invasive blood pressure (IBP and NIBP), SpO2, ventilator and temperature were selected since these constitute the basic monitoring requirements for a patient in the ICU. Alarms from other devices (e.g., intracranial pressure and pulse contour cardiac output (PiCCO) monitor) were discarded. This assures that the alarms are issued from the same number of devices for all included patients. Technical alarms (battery levels, disconnections) were discarded as well. Alarms from devices such as syringe or infusion pumps are not saved in these alarm logs. The alarms were then linked to the respective patients (through the bed name where the alarm was generated and the bed name each patient occupied) and the rate of alarms per 24 h for each patient was calculated. To have uniform data for all patients, each 24-h block started at 6 a.m. (start of the morning shift in these ICUs). An above-median and below-median alarm rate were defined as “high” and “low”, respectively. Alarm rates that were equal to the median were randomly categorized as “high” or “low”.

The variables described above were extracted for patients identified through the alarm logs after inclusion criteria were filtered. The data were grouped into 24-h blocks, all starting at 6 a.m. Continuous variables (such as numerical scores—GCS, RASS, SAPS II—that are measured multiple times per day) were summarized using the median for each patient and day. The CAM-ICU test is generally performed several times per day: The variable ‘CAM-ICU positive’ indicates whether one of the tests during a certain day was positive for the patient. Binary data were summarized to indicate whether a patient received the intervention on a certain day (e.g., was the patient invasively ventilated on this day? YES/NO). The type of mechanical ventilation was categorized as invasive or non-invasive depending on the airway device the patient received.

### Statistical analysis

A univariate analysis was performed using a t-test for continuous variables and a chi-square test for categorical variables. A multivariable logistic regression analysis including the aforementioned covariates was performed. Patient cases with missing values were excluded and the complete-case method was adopted. To test for collinearity between covariates, the variance inflation factor was computed (VIF). The VIF is a metric calculated for each variable in the model. It represents the ratio of the variance of the complete model to the variance of a model containing only that variable. Since all variables had a VIF lower than 5.3, no variables were excluded from the logistic regression model. The significance level was set at α = 0.05.

### Ethics approval and consent to participate

The ethical approval was granted by the Ethics Commission of the Charité-Universitätsmedizin Berlin (Chairman: Prof. Morgenstern, ethics vote number: EA1/127/18, ethics vote date: 10/27/2021). All procedures were followed in accordance with the Ethics Committee of the Charité-Universitätsmedizin Berlin and with the Helsinki Declaration of 1975. Due to the retrospective nature of the study, informed consent was waived by the data protection department of the Charité-Universitätsmedizin Berlin based on the Berlin State Hospital Act.

## Results

### Dataset

The extracted raw alarm logs contained 9,748,329 alarm start entries. After summarizing the entries per day and filtering the patient inclusion criteria, the final number of observations included in the analysis was 26,890, from 3205 different patients. Some of these patients were re-admitted to the ICU, the total number of cases is 3334. The median (interquartile range) alarm rate was 119(169) per patient per day, with a range of 1–9438.

### Group comparison

Table 1 shows the summary and differences between patients in the high- and low-alarm-rate groups. A higher percentage of patients in the high alarm rate group were males and they were on average older than the patients in the low alarm rate group (p < 0.001 for age and sex). There was no difference between the average BMIs of both groups (median 27 for both). Almost half of the patients were admitted as medical, slightly more than a third were surgical emergencies and the rest (14.1% and 15.2% for the low and high alarm rate group respectively) were planned surgical cases. The average patient in the high and low alarm rate groups had severe comorbidities with a CCI mean of 5.8 in the high alarm rate group and a slightly lower mean of 5.4 in the low-alarm rate group (p < 0.001). Patients with a higher alarm rate had more invasive interventions (invasive mechanical ventilation and invasive blood pressure monitoring) and were more likely to have a central venous catheter (p < 0.001). More patients with pre-existing cardiovascular diseases (coronary heart disease, heart failure, hypertension) and chronic kidney failure are in the high-alarm group. The percentage of patients with diabetes is comparable in both groups. There are more patients with asthma (p = 0.005) in the low alarm group, and more patients with chronic obstructive pulmonary disease (p < 0.001) in the high alarm group. Patients with a higher alarm rate had on average a higher SAPS II score (40 vs. 36, p < 0.001) and higher SOFA scores (5.3 vs 6.2, p < 0.001) indicating a higher risk of mortality. They also had a slightly higher yet significant RASS (-1.1 vs -1.2, p < 0.001). There is a higher rate of positive CAM-ICU outcomes (16% vs 18%, p < 0.001) and a slightly lower GCS in the high alarm rate group (11 vs 10 p < 0.001). There was no difference between the rate of dialysis (p = 0.12) or isolation status (p = 0.24), however more patients received ECMO in the low-alarm group (9.2% vs. 6%, p < 0.001).

**Table 1:** Characteristics of patients with a low and high alarm rate.

Variable	Low alarm rate (N = 13,409)	High alarm rate (N = 13,481)	p-value
Age (year, mean ± SD)	61 ± 17	66 ± 15	< 0.001
Sex (M)	8008 (59.7%)	8593 (63.7%)	< 0.001
BMI (mean ± SD)	27 ± 7.0	27 ± 6.6	0.831
Admission type			
Medical	6802 (50.7%)	6645 (49.3%)	0.013
Scheduled surgical	1887 (14.1%)	2047 (15.2%)	
Unscheduled surgical	4720 (35.2%)	4789 (35.5%)	
Charlson comorbidity index (mean ± SD)	5.4 ± 3.5	5.8 ± 3.3	< 0.001
Coronary heart disease (TRUE)	2521 (18.8%)	3163 (23.5%)	< 0.001
Diabetes (TRUE)	5466 (40.8%)	5577 (41.4%)	0.319
Heart failure (TRUE)	2942 (21.9%)	3822 (28.4%)	< 0.001
Chronic obstructive pulmonary disease (TRUE)	1484 (11.1%)	1723 (12.8%)	< 0.001
Chronic kidney failure (TRUE)	2394 (17.9%)	2940 (21.8%)	< 0.001
Asthma (TRUE)	409 (3.1%)	335 (2.5%)	0.005
Hypertension (TRUE)	7796 (58.1%)	8903 (66.0%)	< 0.001
Invasive mechanical ventilation received (TRUE)	6164 (46.0%)	6934 (51.4%)	< 0.001
Invasive blood pressure monitoring received (TRUE)	11,591 (86.4%)	13,120 (97.3%)	< 0.001
Dialysis received (TRUE)	2590 (19.3%)	2708 (20.1%)	0.115
ECMO received (TRUE)	1239 (9.2%)	807 (6.0%)	< 0.001
Isolation status (TRUE)	3112 (23.2%)	3046 (22.6%)	0.237
Central venous catheter placed (TRUE)	7651 (57.1%)	8284 (61.4%)	< 0.001
CAM-ICU score positive (TRUE)	2160 (16.1%)	2425 (18.0%)	< 0.001
Glasgow Coma Scale (mean ± SD)	11 ± 4.4	10 ± 4.2	< 0.001
Richmond Agitation Sedation Scale (mean ± SD)	− 1.2 ± 1.9	− 1.1 ± 1.8	< 0.001
Simplified Acute Physiology Score II (mean ± SD)	36 ± 13	40 ± 13	< 0.001
Sequential Organ Failure Assessment (mean ± SD)	5.3 ± 4.2	6.2 ± 3.9	< 0.001

BMI, body mass index; ECMO, extracorporeal membrane oxygenation; CAM-ICU, confusion assessment method for the ICU.

### Variables associated with a high alarm rate

We fitted a multivariable logistic regression model with these characteristics to assess the independent effect of each characteristic on the alarm rate while controlling for the other variables. The results of the model are presented in Table 2. They show that patients who received invasive blood pressure monitoring (OR 4.68, 95%CI 4.15–5.29, p < 0.001) or invasive mechanical ventilation (OR 1.24, 95%CI 1.16–1.32, p < 0.001), and patients who were previously diagnosed with chronic kidney failure (OR 1.18, 95%CI 1.1–1.27, p < 0.001), heart failure (OR 1.26, 95%CI 1.19–1.35, p < 0.001) or hypertension (OR 1.19, 95%CI 1.13–1.32, p < 0.001) were more likely to have high alarm rates. RASS was significantly associated with a higher alarm rate (OR 1.22, 95%CI 1.18–1.25, p < 0.001). Patients admitted to the ICU due to a planned surgical procedure were more likely to trigger more alarms than patients admitted to the ICU due to non-surgical reasons (OR 1.22, 95%CI 1.13–1.32, p < 0.001), although there was no difference between patients admitted to the ICU due to a surgical emergency and patients admitted in medicine (p = 0.8).

**Table 2:** Independent patient characteristics associated with a high alarm rate.

Characteristic	OR^a^	95% CI^a^	p-value
Age	1.02	1.01, 1.02	< 0.001
Sex (M)	1.06	1.00, 1.12	0.034
BMI	1.00	0.99, 1.00	0.022
Admission type			
Medical	–	–	
Scheduled surgical	1.22	1.13, 1.32	< 0.001
Unscheduled surgical	1.01	0.95, 1.07	0.8
Charlson comorbidity index	0.95	0.94, 0.96	< 0.001
Coronary heart disease (TRUE)	1.01	0.94, 1.08	0.9
Diabetes (TRUE)	0.97	0.91, 1.02	0.2
Heart failure (TRUE)	1.26	1.19, 1.35	< 0.001
Chronic obstructive pulmonary disease (TRUE)	0.91	0.84, 0.99	0.028
Chronic kidney failure (TRUE)	1.18	1.10, 1.27	< 0.001
Asthma (TRUE)	0.91	0.78, 1.06	0.2
Hypertension (TRUE)	1.19	1.13, 1.26	< 0.001
Invasive mechanical ventilation received (TRUE)	1.24	1.16, 1.32	< 0.001
Invasive blood pressure monitoring received (TRUE)	4.68	4.15, 5.29	< 0.001
Dialysis received (TRUE)	0.67	0.62, 0.72	< 0.001
ECMO received (TRUE)	0.51	0.46, 0.57	< 0.001
Isolation status (TRUE)	1.09	1.02, 1.17	0.009
Central venous catheter placed (TRUE)	0.98	0.93, 1.04	0.6
CAM-ICU score positive (TRUE)	0.98	0.92, 1.06	0.7
Glasgow Coma Scale	0.98	0.97, 0.99	0.002
Richmond Agitation Sedation Scale	1.22	1.18, 1.25	< 0.001
Simplified Acute Physiology Score II	1.01	1.01, 1.02	< 0.001
Sequential Organ Failure Assessment	1.07	1.06, 1.08	< 0.001

BMI , body mass index; ECMO, extracorporeal membrane oxygenation; CAM-ICU , confusion assessment method for the ICU.

^a^OR, odds ratio; CI,  confidence interval.

### Variables associated with a low alarm rate

Patients who received dialysis or ECMO had higher odds of a low alarm rate (OR 0.67, 95%CI 0.62–0.72, p < 0.001 and OR 0.51, 95%CI 0.46–0.57, p < 0.001 respectively, Table 2).

## Discussion

Our aim was to identify patient characteristics associated with a high alarm rate in order to emphasize ICU alarm management on patients more likely to have a high alarm rate.

We included only alarms that are associated with the patient's health status; all technical alarms were discarded. To reflect the patient’s health status, we included several variables from different categories (i.e., patient demographics, hospital stay characteristics, clinical scores), and found that invasive blood pressure monitoring, invasive mechanical ventilation, heart failure, chronic renal failure, hypertension, high RASS and scheduled surgical admission were significantly associated with a high alarm rate.

Patients with pre-existing conditions of chronic kidney failure, hypertension and heart failure are usually more unstable and challenging to care for, especially in an acute situation, which would explain the higher rate of alarms compared to patients without these conditions. The same is true for patients who received invasive blood pressure monitoring and invasive ventilation. However, we did not find a significant association of the other diagnoses (i.e., coronary heart disease, asthma) and the clinical scores (i.e., GCS, SAPS II) with a higher alarm rate. This may be explained by a tendency of undercoding diagnoses in EHRs^26–28^. As for patients with a high RASS, the higher number of alarms may be due to artifacts caused by movement or agitation, for example.

Dialysis and ECMO were significantly associated with a low alarm rate. A possible explanation for this counterintuitive finding could be that these patients are usually sedated and intubated, resulting in less alarms related to movement. However, invasive ventilation was associated with a higher alarm rate. As stated above, the alarms of the ECMO and dialysis devices were not included in the alarm rate calculated for the patients in this study.

From these findings, we can suggest that ICU staff should pay closer attention to the alarm management and alarm hygiene of patients with characteristics associated with a high alarm rate. Many solutions to reduce the high rate of alarms have been proposed in the literature, e.g., machine learning algorithms^22^ and sensor fusion methods^23^. However, most of these suggestions have not found their way into clinical routine yet. Nevertheless, we believe that there are many steps that can be taken in order to alleviate the alarm burden in ICUs. Individualizing alarm thresholds for example is an important first step^29^. Although this suggestion might seem trivial, there is a significant number of staff members that adhere to the default thresholds of the monitoring devices and do not adjust the thresholds according to an individual patient’s need^30,31^. This might be either due to lack of time/will or of know-how. Therefore implementing workshops as suggested by Brantley et al.^21^ and Bi et al.^32^, and assigning monitor superusers (staff members that undergo training in and become experts in setting up monitoring devices) as described by Sowan et al.^33^, are also concrete steps that ICU staff can undergo to reduce the high rate of alarms. Another solution is to implement the use of the pause function while examining or performing interventions on patients^34^, or to implement a delay between the deviation of the vital parameter and the sounding of the alarm^35^.

We hope that this study encourages further research directed towards patient-specific effects on monitoring alarms. We believe that developing individualized alarm management for patient monitoring systems will strongly alleviate alarm fatigue and even reduce the rate of false alarms.

This study has several limitations. Although multiple ICUs were included in this study, it remains a single-center retrospective study, utilizing EHRs which are prone to be biased, especially regarding diagnosis^26–28^. Due to the retrospective nature of the study, differentiating between false and true alarms is extremely complex and was not performed. However, only patient-related monitoring alarms were included; technical alarms (from monitoring or other medical devices) were excluded. Furthermore, there is no absolute threshold defining a high alarm rate^36^. We therefore set the threshold as the median alarm rate of our dataset. Future studies should investigate the criticality of alarms on patient level (i.e., yellow, red alarms) as well as other metrics of ICU alarm load such as alarm floods as previously described^37^.

## Conclusion

The high rate of alarms in ICUs is an issue that impairs the quality of patient care, increases the staff’s stress levels, and can even cause sentinel events. This study is the first to analyze the effect of patient characteristics on ICU alarm rates. We showed that invasive blood pressure monitoring, invasive mechanical ventilation, preexisting chronic kidney failure, hypertension, and heart failure, a high RASS as well as scheduled surgical admission can significantly increase the ICU’s alarm rate. ICU staff should pay close attention to alarm management, especially with patients that have these characteristics. Our findings are an important step towards developing novel alarm management strategies incorporating patient-specific characteristics. Future studies with other ICUs should be performed to confirm our findings and examine whether interventions on alarm management targeted at these patients result in a significant reduction of the alarm rate in the ICUs.

## Data Availability

The datasets used during the current study are located in the internal network of the Charité-Universitätsmedizin Berlin and are not publicly available due to the current data privacy policy, but are available from the corresponding author on reasonable request.
